# Facilitators and barriers to communication in rehabilitation services across healthcare levels: a qualitative case study in a Norwegian context

**DOI:** 10.1186/s12913-023-10222-2

**Published:** 2023-12-04

**Authors:** Randi Skumsnes, Hilde Thygesen, Karen Synne Groven

**Affiliations:** 1 Centre for Diaconia and Professional Practice, VID Specialized University, Oslo, Norway; 2 Department of Innovation and Research, City of Stavanger, Stavanger, Norway; 3 Centre for Health and Technology, Faculty of Health and Social Sciences, University of South-Eastern Norway, Borre, Norway; 4 Department of Rehabilitation Sciences and Health Technolgy, Faculty of Health Sciences, Oslo Metropolitan University, Oslo, Norway; 5 Faculty of Health Studies, VID Specialized University, Oslo, Norway

**Keywords:** Interprofessional communication, Information sharing, Rehabilitation culture, Access to information, Unclear responsibilities, Organisational boundaries, Informal communication, Coordination

## Abstract

**Background:**

People with problems in functioning following severe injury or illness often need multiple and combined interventions in their rehabilitation processes. In these processes, communication and collaboration between the involved healthcare professionals are essential. Despite efforts in research and policy, communication across hospital and primary healthcare services and within the primary healthcare settings remains challenging. In one region of Norway, a new intermunicipal rehabilitation team has been established to supplement the traditional services and context-bound research is needed to gain insight into the complexity of the new communication structures that are developing. The aim of this study was to explore facilitators and barriers to communication to inform further improvement of the services.

**Methods:**

A qualitative case study design was used to explore the exchange of patient information in the rehabilitation processes of four patients. Data collection included participant observations in communication situations and an exploration of the electronic patient records of these four patients. Reflexive thematic analysis was used to analyse the empirical data.

**Results:**

The complex rehabilitation processes explored involved a large number of actors across healthcare organisational levels. Lacking a common culture for rehabilitation, poor access to written information and unclear responsibility for sharing information across organisational boundaries seemed to represent barriers to interprofessional communication. Joint meetings, the use of common rehabilitation tools and language and establishing informal communication channels served to facilitate communication.

**Conclusion:**

The intermunicipal team collaborating across different organisational levels added complexity to communication structures, but also facilitated interprofessional communication by promoting formal and informal ways of exchanging information. However, the intricate organisational divisions of healthcare provision in the Norwegian context represent boundaries which can be difficult to overcome. Therefore, cross-organisational coordination services should be developed.

**Supplementary Information:**

The online version contains supplementary material available at 10.1186/s12913-023-10222-2.

## Background

This study revolves around challenges of communication in the rehabilitation of persons in need of multiple and combined interventions. Rehabilitation can be seen as services supporting a person with health conditions to improve or maintain functioning [1]. Whereas rehabilitation research has traditionally aimed at producing knowledge of the effects of interventions [1, 2], there has also been a call for action to strengthen the healthcare systems providing the rehabilitation [3, 4]. In Norway, hospitals and municipalities have separate organisational levels and funding. Acute medical care and early-stage rehabilitation services are provided by regional hospitals, while the municipalities provide primary healthcare services including long-term rehabilitation. Over the past decade, the authorities have promoted more services to be provided in primary healthcare settings closer to where the patients live [5]. Regulations and guidelines for rehabilitation emphasise processes based on coordinated, continuous and evidence-based interventions [6]. Yet an ongoing need exists for improved collaboration between professionals in all the involved healthcare organisational levels [7–11].

Rehabilitation characterised by specialisation and healthcare professionals’ solo work increases the risk of fragmented and ineffective services, especially when a rehabilitation process requires ‘an orchestra of services in which each instrument can blend with the others’ [12]. The dynamics between those involved in interprofessional collaboration is believed to generate better results than the mere sum of each individual part [13], and collaboration and coordination between the healthcare professionals involved are key aspects in rehabilitation [14]. Interprofessional communication means giving relevant information to the right persons to make appropriate decisions based on integrated professional knowledge [15]. Structured, continuing, and timely interprofessional communication has been found to improve patient-centred care, team efficiency and rehabilitation progress, as well as improving patient safety and patient satisfaction [16, 17]. Most research on interprofessional collaboration and communication has been conducted within a single healthcare context, such as a hospital department [18] or a primary care setting [19]. Power imbalance, organisational structures and leadership can be barriers to efficient teamwork in hospitals [20]. Communication methods like regular meetings and open channels and technology for communication have been found to facilitate collaboration [21]. Research on services across healthcare organisational levels has found lack of commitment across organisations, conflicting interests, limited resources, insufficient information exchange and poor coordination of patient pathways forming barriers to the integration of services [22]. Systems emphasising information exchange, education and negotiation between different stakeholders can, on the other hand, facilitate communication in transitions from hospital to primary care [23].

Different strategies have been tried to meet the challenges of collaboration and to strengthen the services in transfers from hospital to primary rehabilitation care. In one region of Norway which is the empirical context of this study, a new intermunicipal team (IMT) was established in 2019. The team is funded by the 15 municipalities in the region. These municipalities differ extensively in size, organisational structure and competence handling patients with complicated and long-term rehabilitation needs. The introduction of the IMT team has involved an extended number of professionals playing a role in each rehabilitation process and the development of new communication structures. Therefore, the complexity characterising the rehabilitation process [24] has increased even more in this regional setting. As developing integrated healthcare services is strongly context-bound [25], research is needed to explore and to gain insight into these new evolving communication structures and processes.

Conceptual frameworks of interprofessional collaboration are built on various theoretical foundations, often related to organisational theory or organisational sociology which consider both the structural and process dimensions of collaboration [26]. In our study, we are inspired by Lauvås and Lauvås’ approach defining interprofessional collaboration as interaction between representatives of different professions aiming to ensure quality of the work and the development of a common basis for knowledge [15]. This approach emphasises four different theoretical perspectives on interprofessional collaboration, namely the social-psychological, the knowledge-sociological and the perspectives of organisations and of professions. Communication is described as one of the core elements in collaboration with its two purposes being the sharing of information and the building of relations [15].

A matrix can frame rehabilitation as an interdisciplinary field in a complex modern healthcare system [27]. This matrix framework is presented in Table 1 and encompasses three groups of agents in the rehabilitation process: individuals with problems in functioning, the professionals providing the services and the governmental authorities. Furthermore, this matrix involves how these groups are positioned and act on the micro-, meso-, and macro-levels. For research purposes, the matrix can be used to contextualise relevant rehabilitation studies.

**Table 1:**
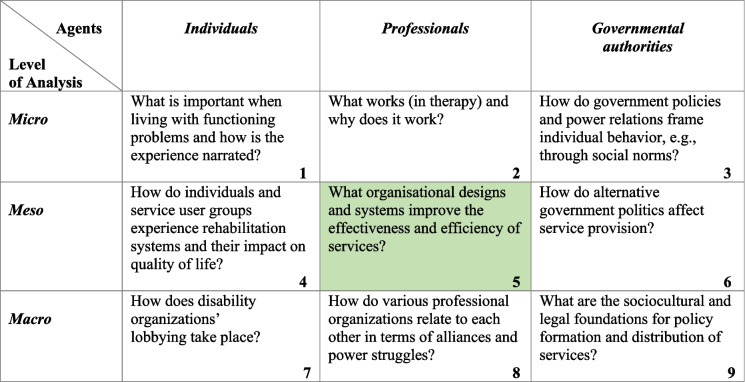
The matrix of agents and levels of analysis in producing rehabilitation knowledge

The matrix by Solvang et al. presents examples of key research questions relevant to rehabilitation. We have highlighted cell number five as we consider organisational designs like rehabilitation chains and collaboration to be of particular interest to explore in our study [27]

In planning our study and defining research questions, our focus aligns primarily with the fifth cell of the matrix. New knowledge of communication amongst the involved professionals can contribute to further improving the rehabilitation services in this region of Norway.

### The aim of the study and research questions

The aim of this study is to explore facilitators and barriers to communication in the rehabilitation process for patients receiving complex rehabilitation services. We address the following two research questions:

- RQ1: How do the involved professionals exchange information in the transition from hospital to primary care, within the municipality services, and with the patients?
- RQ2: Which factors facilitate and contribute to communication in the rehabilitation process and what are the barriers to communication?

## Methods and materials

### Study design

To explore communication in rehabilitation processes and study it in interactions within its actual context, a qualitative case study design was chosen. A qualitative and interpretative case study is relevant when examining a part of a whole, seeking patterns and understanding of the interrelationships among the parts, to present assertions and to stimulate the reader to further reflections [28]. With an interpretivist case study approach, we explored the individual and shared meanings from different perspectives [29]. Using this interpretivist case study approach we regard knowledge being constructed and not discovered. We thus provide descriptions and interpretations of the communication in the cases, allowing readers to consider whether the findings are relevant to their own context [28].

### Defining the case and the study context

The case explored in our study is the ongoing information exchange in the rehabilitation process of neurological patients. Specifically, these processes involved services provided by one regional hospital, some of the municipalities in the region, the IMT team, and some private and semi-private healthcare actors. How to share responsibility, and how to collaborate in the transitions between these organisational levels of services are regulated through laws, national guidelines and regional agreements [6, 10, 30–33]. The municipalities have organisational variations due to size, geographical and demographical variations. However, all the municipalities contain separate departments with designated employees, managers and defined economical frames, providing rehabilitation services for inpatients in nursing home rehabilitation departments and in home-rehabilitation services.

As mentioned in the introduction, unique to the study context is the multidisciplinary and intermunicipal team IMT which was recently established with an aim to improve transitions from hospital to primary care. The IMT provides a supplement to each municipality’s rehabilitation services as these are struggling to offer interventions of sufficient intensity. Some municipalities also lack the competence to handle the most demanding cases, especially complex neurological cases. The IMT includes a physiotherapist (PT), an occupational therapist (OT), a psychologist, and a medical doctor, all of whom have specialised rehabilitation competences. A team manager is responsible for the team and for clarifying IMT involvement with managers in the current patients’ home municipality. When the IMT becomes involved in a patient’s case, the professionals in the team early on establish contact and start collaborating with hospital staff and staff in the municipality. Whilst the patient is still hospitalised, rehabilitation potential and service prospects in the home municipality might not be settled, making it challenging to plan for discharge. The IMT follows the process closely and assists in connecting relevant professionals across the different healthcare organisational levels. The team promotes using common tools for goalsetting and evaluating the rehabilitation process. To reduce the information gap between hospital, IMT and general practitioners in primary care, the IMT’s medical doctor was recruited from the hospital and continues to work there as well as in the IMT team.

### Recruiting cases and participants

Inclusion criteria for the cases included in the study were hospitalised adult persons in need of specialised long-term neurological rehabilitation services at the municipality, including IMT services. To gain a wide insight into the processes, we strove to include a variation of patients in terms of age, gender, neurological diagnoses and rehabilitation needs and the size of their hometown/municipality. The staff at the regional hospital rehabilitation department and the IMT were informed about the study and were requested to assist in recruiting patients. The staff briefly informed patients who met the inclusion criteria of the study and asked them to consider participating. For patients who responded positively, the staff provided contact with the researcher and formal inclusion in the study was made. Inclusion involved informing the patients more thoroughly about the study, both orally and in writing. In addition, the patients were informed of their right to withdraw their consent at any time. Four patients were recruited over a period of ten months. As the purpose of the study was to follow the ongoing communication along the rehabilitation process, all persons directly involved with the participating patients also became involved in the study. Therefore, close family and the healthcare professionals involved with the patients were informed about the study and were also asked for their consent to participate.

The staff assisted in recruiting participants over time. The total number of patients invited to participate and the number of patients declining the invitation were not reported.

### Data collection

Data were collected through participant observations [34] in communication settings and from electronic patient records (EPRs). The first author conducted participant observations in different settings, including hospital meetings for discharge planning, information and knowledge transfer meetings, and listening to communication between the patient and healthcare professionals in goalsetting, planning, and during the interventions. The researcher mostly observed from the sideline, and when appropriate, she participated in informal conversations. Occasionally, the researcher asked the patients, relatives, and professionals questions to elaborate on topics arising during the observations that were of special interest to the research focus of the study. In addition to participant observation, the first author had access to the participants’ EPRs in their home municipality to study documented written digital communication between the involved healthcare professionals across and within the organisations. Supplementing data from participant observation with EPRs was done to ensure a broad insight into the process of rehabilitation, as the researcher was not able to physically observe all communication taking place within each case.

To structure the observations and data collection from the EPRs, observation guides developed for this study were used (see Supplementary file 1). The main issues of interest in the data collection were the exchange of relevant information about a) the patient’s rehabilitation status, goals and needs; b) the healthcare professionals’ negotiations related to the distribution of responsibilities for interventions; and c) the coordination of the rehabilitation interventions. The handwritten notes from the participant observations and EPRs were transcribed into de-identified fieldnotes per patient case on a computer and stored in a secured database. The names of the participants were coded and stored separately.

As the participant observations were time-consuming, and obtaining access to participate in the ongoing practice demanded flexibility [34], only one or two cases were followed at the same time. The period for fieldwork of each case varied from four to eight months. The EPRs were examined parallel to the observations as well as afterwards.

### Analysis

The data material was first summarised providing a descriptive presentation of the complexity of all involved, approaching the first research question of how these involved actors were exchanging patient information. The analytic approach for the research question on facilitators and barriers to communication followed the steps of reflexive thematic analysis by Braun and Clark [35]. In step one, the first author read all the written fieldnotes from the four cases several times to become more familiar with the data. In step two, the first author made a preliminary open analysis tagging the segments in the data material of each case, reducing the details and suggesting code labels. Tables in Microsoft Word were used to structure the material and the codes. Examples of some of the preliminary codes were ‘acting open and respectful’, ‘creating security and trusting relations’, ‘unpredictability in the patient’s situation’, ‘different degrees of flexibility and autonomy in planning the work day’ and ‘time to go into details’. All authors discussed the preliminary codes looking for patterns across the cases. Themes covering material from all cases were suggested by the first author in step three, and all authors discussed these themes in step four. Then the themes were revised in an abductive and reflective process including all authors considering previous research and personal experiences, going back and forth between the data material and the themes.

The preliminary findings were also presented to a group of rehabilitation professionals to see if they were familiar with the material and could relate to the themes, as a member checking [36]. Their feedback helped us to adjust some of the codes and themes.

### The researcher’s role

The study was informed by the first author’s experiences as an occupational therapist working for 20 years in primary healthcare, having knowledge about the field and effective access to the setting professionally, organisationally and socially. This necessitated considerations of the ‘insider’ perspectives regarding, e.g., presumptions or the possibility of not noticing elements taken for granted. Attention was paid to how the studied phenomenon was interpreted through the researchers’ lenses, how the field had an impact on the researchers and how this affected the study setting [34]. Researchers’ subjectivity in qualitative studies can be a resource in conducting analysis, and reflexivity involves reflecting on assumptions, expectations, choices and actions [35]. A log was maintained for noting these reflections along the process.

### Ethical considerations

The project was approved by the Regional Committee for Medical and Health Research Ethics (REK) West (REF, 239,895) and by the involved organisations’ data protection officers. The patients, close family and the directly involved healthcare personnel were presented with the study contents and gave their written consent to participate in the study, permission to collect and store data concerning them and to publish the findings.

## Results

To provide a picture of the complexity and the large number of healthcare departments, professions and persons involved, we start this section by describing the cases and the communication methods used for the exchange of patient information. Next, we present our results exploring facilitators and barriers to exchange of patient information.

### The cases

Living in three different municipalities, we included four patients in their rehabilitation process following severe neurological injuries or illness. In Table 2, we present the patients collectively to protect them from being recognised. The departments and professions presented in the table were those most involved in providing healthcare services during the patients’ rehabilitation processes. A variety of people representing the different healthcare professions were involved in the cases, but not every listed department was involved in all four cases.

**Table 2:** The patients, departments and professionals included in the study

**Characteristics of the four patients represented in the cases**		
Ages	41–68 years	
Genders	Female	1
	Male	3
Neurological condition and background for the need for rehabilitation services	- Multiple sclerosis (had the diagnosis for several years, but now with new problems in functioning)	1
	- Parkinson’s disease (had the diagnosis for several years, but now with new problems in functioning)	1
	- Stroke (recently diagnosed)	2
Patient living situation	Living alone	2
	Living with partner/spouse	2
Referred to municipality and IMT from	Inpatient rehabilitation department in regional hospital	3
	Outpatient rehabilitation department in regional hospital	1
Transferred from hospital to	Home (the patient’s own apartment or house)	2
	Inpatient rehabilitation department in municipality, then home (the patient’s own apartment or house)	2
Size of home municipality of the patients	10,000–50,000 inhabitants	2
	Over 50,000 inhabitants	2
**Departments providing healthcare services in the cases**		
Departments in hospitals	Rehabilitation department in regional hospital (in- and outpatient)	
	Other departments in regional hospital (geriatric, neurological, etc.)	
	Specialised national hospital department (neurological)	
Departments in the municipalities (primary health and care services)	Inpatient rehabilitation departments in nursing homes	
	Case managing offices	
	Home nursing departments	
	Home rehabilitation teams	
	Mental health teams	
	Occupational and physiotherapy departments	
	Special pedagogical department for speech therapy, vision and hearing	
	Intermunicipal rehabilitation team (IMT)	
Departments in private practices or semi-private practice	General practitioners (GP)	
	Speech therapy	
	Physiotherapy	
	Center for prosthetics and orthotics	
	Firms providing ‘user-controlled personal assistance’	
Departments, government funded	NAV (Norwegian Labour and Welfare Administration)	
	- Benefits and welfare services	
	- Assistive Technology Center	
**Professionals/disciplines providing services in the cases**		
Healthcare professions	Physiotherapists (PT)	
	Occupational therapists (OT)	
	Medical doctors/physicians (MD)	
	Registered nurses	
	Mental health nurses	
	Nurse assistants	
	Speech therapists	
	Psychologists	
	Case managers	
	Social workers	
	Orthopaedic engineers	
	Personal assistants	

An overview of the participating patients, departments and healthcare professions involved in the rehabilitation processes observed in this study

### Communication methods and magnitude of the information exchange

Healthcare professionals from all departments involved communicated directly with the patients to give and receive information. This included oral communication either in person, via phone calls or in physical or digital meetings, as well as written communication, such as digital messages (text messages or email) and printed reports.

The healthcare professionals communicated across the organisational healthcare levels of hospital departments, municipality departments, the IMT and others. Personnel also communicated across departments within the municipality services and across different parties involved within the same department. Communication took place between persons practicing the same profession as well as across disciplines. There was formal communication in planned meetings, written electronic reports sent between departments and informal communication, such as face-to-face conversations, phone calls or short written electronic messages sent between two to three of the involved parties.

The healthcare services provided were documented in the patients’ EPR. The hospital had one enclosed EPR system, and each municipality had its own enclosed system. The three municipalities represented in this study used three different EPR systems. The IMT documented their practices in one of the municipalities’ EPR systems. In addition, we found that the GPs had their own record systems. Healthcare professionals at hospital, the municipalities and at the GPs’ offices did not have access to each other’s EPRs, but it was possible to send secure electronic messages and reports between these systems. Healthcare professionals in private practice and pedagogical professionals had their own systems for documentation. These systems were unable to digitally communicate with the systems at the hospital or in the municipality, and paper reports were sent to share information.

Within each municipality, the EPR systems consisted of several sub-records linked to each discipline, such as nursing, physiotherapy and case management. The three EPRs used by the municipalities were constructed differently with regard to how the professionals could access other professions’ sub-records. In one system, several professions could read each other’s daily reports keeping an overview, but in the two other systems this was not possible. Written information or reports received from hospital to the municipality EPR were not automatically distributed to all the different professionals within the municipality involved in the case at hand; rather, they had to provide access to other professionals in order to share information.

**Fig. 1:**
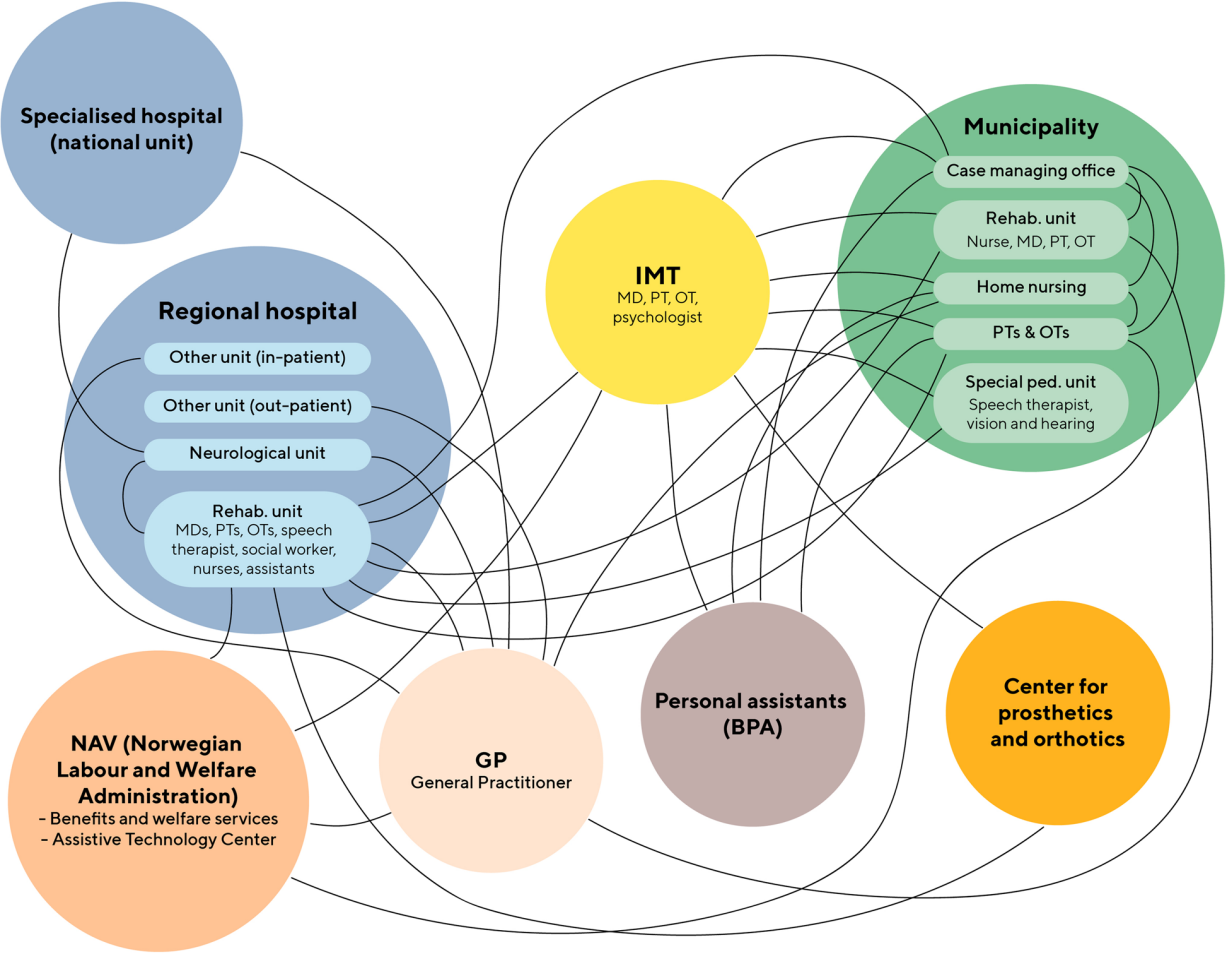
Involved departments and professions and their communication in one patient’s case. The lines represent communication observed between healthcare professionals in one person’s’ rehabilitation process, such as oral, written, electronic and physical information exchange. (Size, colours and placement of the main circles and text do not represent any quantitative or hierarchical meaning; these are due to practicalities in designing the figure.)

To illustrate the complexity and magnitude of the information flow, Fig. 1 shows the communication observed in one of the patient cases. Please note that to simplify the graphic, we have left out all direct communication between the professionals and the patient which was taking place parallel to the communication between the professionals.

Our findings suggest that the complexity regarding the number of departments and professionals involved, including the different methods for sharing information, might increase the risk of not obtaining an efficient information flow in terms of getting relevant information to the right person at the right time. Additionally, in the case represented in the above figure, and in two other cases, we did not find that a dedicated professional was maintaining an overview of the information or providing overall coordination, although a case manager coordinated some of the municipal services in the fourth case. Structured tools, like an individual care plan (ICP) or an electronic individual care plan (e-ICP), were not used to coordinate any of the patients’ follow-ups in the four cases observed. Overall, we found several challenges with the information exchange related to negotiation and allocation of responsibilities for tasks and interventions across the involved parties.

### Facilitators and barriers to information exchange

To study the challenges of information exchange further in our analysis, we explored facilitators and barriers to information exchange in the cases observed. Through our analysis, we identified three main themes: ‘common culture for rehabilitation’, ‘access to written information’ and ‘authority to clarify responsibilities’. In the following sections, we will present each theme and provide fieldnote extracts from situations underpinning the themes.

#### Common culture for rehabilitation

Our findings indicated that having a common understanding and culture for rehabilitation facilitated information exchange, whereas the lack of a common understanding among the different healthcare professionals providing services could serve as a barrier to information exchange. The following fieldwork excerpt from a meeting illustrates how professionals from different professions and healthcare services at the hospital and the municipality worked together. The purpose of the meeting was to examine the patient’s status and needs after discharge from hospital, to plan and adjust further follow-up interventions and to decide how to share responsibilities and coordinate tasks.

*The meeting was with the patient in his home. The professionals participating were a medical doctor, a PT, and an OT from the rehabilitation department at the hospital, a PT from IMT and an OT and a PT from the home municipality. The dialogue in the meeting revolved around topics from a checklist used by hospital professionals, and the Patient Specific Functional Scale. These tools were focused on letting the patient describe his present condition, as well as activities and goals that were important to him.*

The professionals involved in this situation seemed to be familiar with the tools used. This provided them with a common language and an understanding of the rehabilitation process depending on having clear and shared information on which to base further interventions. Another aspect in this situation, was that the professionals from the hospital, municipality and IMT met at the same time. They could potentially have had separate meetings with the patient in his home, exchanging information written or orally afterwards. However, they prioritised a joint meeting. This indicates how highly they valued the simultaneous dialogue with the patient and the other professionals involved in the continuous rehabilitation process.

Throughout our empirical material, we identified several examples of this direct method of exchanging patient knowledge and information, which seemed to facilitate communication. Professionals from the rehabilitation field with a similar background, mostly therapists, often met in person with other professionals and the patients to have a direct and detailed clinical handover. However, there were also professionals who did not attend these interprofessional or clinical handover meetings, arguing that this was too time-consuming and their attendance was not required for them to provide their services. Our reflections are that these differences in practices can relate to healthcare professionals viewing their work and contributions to the patient follow-up in different ways. For example, those providing separate interventions may not consider these to be interwoven or connected with interventions from other professionals and might not experience a need to exchange information or ensure an interprofessional approach. As other healthcare professionals clearly had a different take on this situation, these variations in professional orientation may be seen as a potential barrier to information exchange and collaboration.

#### Access to written information

We found that getting physical and timely access to written patient information was problematical and served as a substantial barrier to communication and information exchange. To ensure safe and appropriate services, healthcare professionals need patient information from other departments and professionals to plan and conduct their interventions. When this information is not gained directly by participating in physical or digital interprofessional meetings or by written reports, it was difficult to obtain the relevant information from the patients’ EPRs. Here is an example from our material illustrating this challenge:

*It was not possible for the private practice PT in the municipality or the PT from the IMT to look up the written patient records from hospital physiotherapy treatment or other detailed medical information from hospital doctors. A short, written, discharge report did not cover all the essential information to plan and adjust the treatment and exercises for the patient at home. Phone meetings with the hospital departments to gain more information was not always possible and it delayed the rehabilitation progress.*

Situations like this were challenging due to the organisational barriers of the enclosed EPR systems. However, as the medical doctor was employed at the hospital rehabilitation department as well as at the IMT, she had access to hospital records and could provide relevant information sharing it with colleagues on the IMT team and serving as a facilitator for information exchange.

Poor access to written information was also an issue *within* each municipality. Another excerpt from our empirical material illustrates challenges with lack of, or poor access to written information. The situation described was an interprofessional meeting at a patient’s home. Present at the meeting was the patient and his spouse, two PTs, an OT and a nurse assistant, all professionals from different departments of the municipality and the IMT. The agenda was to share knowledge and information regarding the ongoing interventions and to settle some goals and plans for further follow-up.

*During the meeting, an intervention initiated by other healthcare professionals earlier in the patient’s rehabilitation process was questioned by the spouse and patient. Apparently, neither of the healthcare professionals present at the meeting knew why this intervention had been initiated and this information vacuum seemed to create insecurity and tension. The spouse expressed that the intervention had not been discussed with them and, if carried out, would influence them negatively. The intervention would also involve professionals from different disciplines and municipal services, who were present at the meeting, without them being able to explain why it was required.*

*As it turned out, after the meeting, the intervention was documented and justified in a brief note in a sub-record in the municipality’s EPR system, as one department at the municipality had received information about the intervention follow-up from the hospital before discharge. However, this information had not been shared with the other municipal professionals involved in the case. Neither had it been shared with or noticed by the professional present at the meeting representing the department where it was documented.*

The fragmented structure of the municipality’s EPRs with sub-records for documenting different but interrelated healthcare services from various disciplines, made it difficult to share and find relevant information across professions. Our interpretations of these situations are connected to how these barriers to accessing written information seemed to make it difficult to plan interventions. In addition, this increased the risk of misunderstanding and insecurity for patients and professionals involved, while also causing unnecessary delays in the rehabilitation process.

#### Authority to clarify responsibilities

Through our analysis, we found a third barrier to sharing and exchanging information, which we associated with unclear and fragmented use of authority. The rehabilitation services observed were often chains of interventions which depended on common efforts and information on decisions from healthcare professionals having different roles in the process. These professionals were employed in departments at various healthcare organisational levels, with separate managers and budgets, and there was no overall authority to determine and coordinate the services and interventions. We have extracted two situations from our observation fieldnotes to exemplify this. The first episode was related to an upcoming discharge from hospital to the municipality of a patient with severe and complex needs.

*It was not yet decided if the patient was to be discharged to an inpatient rehabilitation department at the municipality or directly to his home. Therefore, two OTs and two PTs were potentially responsible for the municipality follow-up, representing the PT and OT home services and the inpatient rehabilitation department. In addition to these four, an OT and a PT from the IMT were involved, as well as an OT and a PT from the hospital rehabilitation department. Not all these professionals were meeting at the same time, but some of them met physically, two or three together with the patient, to plan the transfer and hand over clinical patient information relevant to further follow-up. They also exchanged information using electronic messages or phone calls. Several issues were addressed and planned for, but an essential intervention, the patient’s need for a wheelchair, was not initiated by any of these eight therapists or by any of the other professionals involved.*

According to our reflections on this episode, the many different interventions planned for were not coordinated by anyone with a complete overview of the case. It was not routinely determined which department or profession was responsible for applying for technical aids, leaving the need for action unsolved. This *delayed* the process for weeks before someone realised that the wheelchair had not been applied for.

Another situation related to unclear authority and information exchange was a digital video meeting to plan the discharge from a hospital rehabilitation department to the municipality. There were four hospital professionals attending, two from the municipality and one from the IMT, in addition to the patient and spouse. Hospital staff and the patient and spouse were sitting in a large conference room at the hospital, and the municipal staff and IMT professional attended digitally, either from their own department desks or their home office due to COVID-19 restrictions making it impossible to meet in person.

*The patient and spouse expressed their concerns about further follow-up, describing their previous bad experiences with services from the hospital and the municipality. Therefore, they insisted on an extension of the hospital stay until they could be promised the same quality of rehabilitation services at the municipality as they received at the hospital rehabilitation department. The professionals representing the municipality at the meeting could not guarantee this. Nor were they able to give any promises at the meeting about the details of the follow-up after discharge, as they had to discuss the availability of different services within their municipal departments.*

This episode highlights several challenges in the information flow on allocation of services and decisions in rehabilitation processes. It shows the frustration felt by the patient and spouse in not being provided with the information and security they needed in the discharge situation. The municipality representatives at the meeting were not enabled to provide the patient with detailed information about what to expect of follow-up services. The professionals were acting on behalf of separate departments. The different departments have their own budgets and have to internally prioritise their resources concerning budgets, available professionals, and other patients in need of their services before they can assign interventions in this specific case. Another challenge was that the hospital rehabilitation department provided recommendations for further follow-up after discharge but was not in a position to guarantee the patient these services in the municipality. No overall authority was prepared to provide a coherent service or to help the patient in tying together the information about a clearly fragmented set of services.

We did, nevertheless, observe several situations where professionals developed respectful relations and a trustful atmosphere for information exchange. Such situations included professionals taking informal responsibility for holding on to- and passing on- information to other professionals. These actions facilitated communication and were often seen in situations in which the professionals in the municipality or the IMT knew the patient and the situation well. When it was unclear or undecided who among the municipality staff would be involved, the IMT professionals often collected and retained patient information from the hospital, until it was made clear who would be in charge going forward.

## Discussion

Above, we have presented facilitators and barriers to information exchange structured through three themes: common culture for rehabilitation, access to written information and authority to clarify responsibilities. In the following section, we will discuss these findings in relation to central concepts of interprofessional communication, related research literature and relevant policy documents.

### A paradox in interprofessional rehabilitation practices

Our findings suggest that a variety of assumptions and cultures for rehabilitation exist amongst the involved professionals and that this lack of uniformity could serve as a barrier to communication. A wide range of professionals from the hospital, the municipal departments, the IMT and others were involved in information exchange and collaboration in the cases. In each case, several of the professionals had not worked together before and did not know each other. We consider such relations as challenging in terms of building a common understanding of rehabilitation and for collaboration and teamwork. Common to a wide range of conceptual frameworks for interprofessional collaboration is the concept of ‘sharing’ which involves shared professional perspectives, values and philosophy but also as shared decision-making and responsibilities, sharing plans and interventions, and sharing data [26]. The quality and effectiveness of interprofessional collaboration in multidisciplinary rehabilitation teams have been shown to benefit from regular meetings and from sharing information, knowledge and skills [24], supporting our findings of joint meetings and the use of common tools facilitating communication. Poor collaboration characterised by a lack of common understanding, can be understood in different theoretical perspectives. In a knowledge-sociological perspective, it could be explained by how the different disciplines start forming separate knowledge bases in professional education. In practices where healthcare personnel mainly work closest with colleagues of the same profession, divisive ways of thinking may further develop [15]. Applying a social-psychological approach, lack of clarification of roles and role expectations may challenge interprofessional collaboration and impede the development of shared understanding and assumptions [15].

Interprofessional practice can also be nuanced into interprofessional teamwork, interprofessional collaboration, interprofessional coordination, and interprofessional networks [37]. Interprofessional *teamwork* is associated with close collaboration in fixed and stable groups, while interprofessional *collaboration* and *coordination* are described as ‘looser’ forms of interprofessional work. Interprofessional *networks* are presented as a substantial number of healthcare professionals engaging in one patient case, changing over time as the patient’s needs change, making it dynamic and unlikely for all to meet face-to-face. In a network type of cases, it is not realistic to have collective training and shared team identity among all those involved. A network type of interprofessional practice is described as being the most suitable in predictable, non-complex and non-urgent cases [37, 38].

According to our findings, as Fig. 1 illustrates, a neurological rehabilitation process involves a large number of professionals and considerable ongoing communication related to a number of different aspects of the follow-up. All the cases observed were different in terms of patient’s needs, who was involved, and at what time they were involved in the process. The processes can, therefore, best be described as unpredictable and complex. This recognition illuminates a paradox and a great challenge in making interprofessional collaboration practice feasible in rehabilitation processes. Policy documents emphasise rehabilitation practices involving close collaboration in terms of interprofessional efforts of shared planning, interventions, and evaluation of the services [6, 10] reflecting the factors of close interprofessional teamwork described above. However, the current practices highlighted in our findings were mostly arranged as large and loose networks with unlikely prospects of establishing a common rehabilitation culture across all involved.

### The complexity of digital information structures

Our findings indicate that decisions, collaboration and rehabilitation progress are often delayed due to poor access to essential written patient information across organisational levels and departments. These findings relate to previous research reporting a lack of appropriate technology and system incompatibility to be logistical barriers to effectiveness in communication between hospitals and primary care [22, 39]. Moreover, a national report on EPR systems in Norwegian municipalities has pointed out how this lack of standardisation and the complexity of system structures make information difficult to handle and share [40]. Digital information exchange can also be affected by the healthcare professionals’ use, their preconditions for using such tools, including their digital competencies [41] and, as we found in our study, various perceptions of the need to communicate and share information. Acting on these different views may be related to autonomy. Professional autonomy may contribute to enabling and allowing personnel to make independent decisions, but extensive autonomy within disciplines may also be seen as a challenge to interprofessional collaboration [15].

An electronic individual care plan (e-ICP) is a digital collaboration tool for accessing relevant patient information across the boundaries of organisational levels. This is a web-based tool where the patient and healthcare professionals across different departments and organisational levels can collaborate digitally to plan and document a rehabilitation process using an encrypted log-on [42]. Persons in need of coordinated healthcare services in Norway have a legal right to be provided with an individual care plan [6], in either an electronic version or a paper version. In our study, however, we did not find any use of digital tools for coordinating or accessing written information across healthcare levels. Implementing such new communication tools can meet challenges of technical, organisational and social issues [43]. For example, a Norwegian study showed that implementation and use of e-ICPs was impeded by practical limitations, insufficient time and preferences for more familiar and personal ways of communicating, like telephone or email [44]. Despite strong policy imperatives, ICPs have remained significantly underused in Norway. This underuse is suggested to be related to an ambiguous understanding of rehabilitation and the municipalities’ self-government of how to organise their services [45].

### Formal and informal authority structures

We found challenges of fragmented organisational levels and authorities and unclear responsibilities to be barriers to communication affecting information exchange. These obstacles raised difficulties for professionals in terms of coordinating the services and interventions provided by multiple professionals representing different departments. In Norway, legislation and guidelines on rehabilitation determine the allocation of responsibilities and funding [6, 10]. There are also regional agreements between hospitals and municipalities with directions for collaboration, for example, concerning patient information exchange, responsibility for providing ICPs, and for ensuring coordination. The formal structures of authority are based on these regulations, but we found little cross-organisational coordination and shared decision-making in our observations. According to Lauvås and Lauvås’ organisational theoretical approach to interprofessional collaboration, organisations are social systems framing the interaction between the involved entities. In these social systems, elements like the organisations’ goals, structures, formal and informal power, and communication methods are of significance to collaboration [15]. The involved organisations in our study seemed to share the overall goal of rehabilitation, but as our empirical material shows, this was not operationalised by the organisations regarding structures and authority to enable the professionals to work smoothly together. These findings can be related to a previous study illustrating that allocations of rehabilitation interventions were made in the individual lines of authority, rather than sharing information between departments, and were, therefore, characterised by a lack of joint decision-making [46]. However, as reported in the result section, we also observed informal structures developing and professionals taking responsibility to ensure important patient information exchange. Previous research has also found that professionals actively contributed to interprofessional collaboration by bridging social, physical and task-related gaps in the systems and by negotiating overlaps in roles and responsibilities [18]. Similarly, our findings suggest that the informal and interpersonal actions may contribute to overcoming some of the challenges of unclear authorities among the involved professionals across healthcare levels. Nevertheless, the new collaboration structures including the IMT team, might benefit from a clarification among hospital, municipalities and other involved organisations on how to handle the development of these informal structures.

## Study limitations and strengths

This research has used a case study design situated in a regional Norwegian context and the small sample may raise questions regarding the trustworthiness of the results. Case studies might not be suitable for making grand generalisations [28], but knowledge from case studies can be transferable to other settings and, therefore, make contributions to the fields in question [47]. With a thorough description of the cases and context, we provide the reader with the possibility of considering transferability to another context. We also argue that our findings regarding the facilitators and barriers to communication correspond with previous studies, suggesting that our findings may be of interest for use in other settings.

The interpretative and reflexive analysis makes the study difficult to reproduce and this can be seen as a study limitation. However, we have provided a description of our methods for recruiting participants and data collection, as well as the steps of the analysis and the researcher’s background and involvement, to contribute to transparency [29]. We also provided some of the participants with preliminary findings to check their opinions of the accuracy of the materials.

Another study limitation can be related to data collection. For example, we were not able to access all activity of communication and information exchanges through direct observations of the included participants. In addition, some healthcare professionals declined to participate in the study. Therefore, we omitted data involving these professionals from the analysis. This might have led to an incomplete or distorted picture of all the communication and interaction taking place. However, to complement the observations, we added analysis of the electronic patient records and had informal talks with the participants as a triangulation validity strategy. We also used an observation guide during the observations and to inform us in generating the fieldnotes.

We considered the case study approach to be a suitable design for exploring the phenomenon of communication in rehabilitation in its real context. Few other studies have explored cross-sectional healthcare communication, especially related to new communication structures developing in the context of the Norwegian neurological rehabilitation field including a new intermunicipal team.

## Conclusion

The new IMT team supplementing traditional primary care rehabilitation services seemed to add complexity to the collaboration and communication structures. However, the team also facilitated formal information exchange by promoting the use of joint meetings and common tools. Team members having access to written patient information at the hospital as well as in the municipality also contributed to improved communication. In addition, the informal collaboration and personal commitments also seemed to facilitate interprofessional communication. However, these facilitating efforts were not sufficient to overcome the barriers to information exchange related to the lack of a common culture for rehabilitation, poor access to written information and unclear responsibilities across organisational boundaries. Taken together, our findings point to a discrepancy between authority expectations and the actual structural conditions for interprofessional communication required to provide integrated services.

### Implications for practice and further research

Decision-makers, managers and healthcare professionals need to be aware of the barriers to interprofessional communication revealed by this study. The facilitators presented must also be acknowledged and should inform efforts made to strengthen the rehabilitation services. Managers need to build working environments characterised by a culture of sharing knowledge and information across organisational levels. Also, it is necessary to promote the use of formal and informal communication structures. As the intricate organisational division of healthcare providers involved in rehabilitation services will continue to represent boundaries to collaboration, developing coordination services enabled to act across organisational levels is essential.

Future research should engage in exploring how healthcare professionals experience the barriers to interprofessional communication and how they can handle these challenges in their day-to-day work. Efforts should also be made to explore how the facilitators to interprofessional communication suggested in this study could be disseminated and further developed in healthcare practices.

### Supplementary Information

**Additional file 1.** Observation guides used in the study.

## Data Availability

Restrictions apply to the public availability of the collected data; consent was given strictly for use in this current study. However, the data will be kept for five years and may be obtained from the corresponding author (RS) upon reasonable request.

## Abbreviations

IMT: Intermunicipal team
PT: Physiotherapist
OT: Occupational therapist
GP: General practitioner
EPR: Electronic patient record
ICP: Individual care plan
