# Supplementary material for: Facilitators and barriers to communication in rehabilitation services across healthcare levels: a qualitative case study in a Norwegian context
Source: BMC Health Serv Res. 2023 Dec 4;23:1353. doi: 10.1186/s12913-023-10222-2 (PMC10696812; doi:10.1186/s12913-023-10222-2)
Supplement: Supplementary file 1 — Additional file 1. Observation guides used in the study. [file 12913_2023_10222_MOESM1_ESM.docx]

**Supplementary file 1.**

**Observation guides used in the study**

For the data collection, we developed a five-point checklist as a brief guide for structuring the participant observations. We also developed a short guide for the assessment of patient records in the study. Both these guides are based on the research question, the study protocol description approved by the ethical committee on restrictions regarding patients’ privacy policy, and the Norwegian legislation stating the health care personnel’s obligation to provide patient records of “*… essential and relevant documentation in order to deliver health care of good quality including effective and good patient pathways*”.

***Guide for participant observations***

1. Agenda for the meeting or other collaborative situations observed.
2. Who are the involved actors and what are their roles.
3. What kind of information is shared – and between which involved actors:
   1. Patient status, goals, and needs?
   2. Ongoing interventions relevant to other involved actors?
   3. Relevant rehabilitation knowledge?
   4. Other?
4. Negotiation and distribution of responsibility for interventions and tasks:
   1. Discussions on what is the next step in the rehabilitation process.
   2. How the involved agree on who does what.
5. Methods and tools used in the communication:
   1. Verbal dialogues?
   2. Structured schemes or forms?
   3. Digital tools, film/video, pictures?
   4. Other?

***Guide for assessment of patient records***

What electronic communication methods are used to exchange relevant information in rehabilitation transitions? How is information about these issues shared:

1. Information about the patients’ status, situation, rehabilitation needs and goals.
2. Information about discussions and decisions on how to share responsibility for interventions and tasks.
3. Information on coordination of the services.

Additional elements:

- Timeframes in communications on specific topics, interventions - examples
- Who sends information to whom, and how do they respond to each other?
- Who has access to the shared information?
- How is information formulated and presented?
- Similarities and differences between how the involved communicate within and across health care levels, within its own profession and between professions etc.

[Siter kilden din her.]
